# OGRE: Overlap Graph-based metagenomic Read clustEring

**DOI:** 10.1093/bioinformatics/btaa760

**Published:** 2020-09-01

**Authors:** Marleen Balvert, Xiao Luo, Ernestina Hauptfeld, Alexander Schönhuth, Bas E Dutilh

**Affiliations:** Life Sciences & Health, Centrum Wiskunde & Informatica, Amsterdam 1098 XG, The Netherlands; Theoretical Biology & Bioinformatics, Utrecht University, Utrecht 3512 JE, The Netherlands; Department of Econometrics & Operations Research, Tilburg University, Tilburg 5000 LE, The Netherlands; Life Sciences & Health, Centrum Wiskunde & Informatica, Amsterdam 1098 XG, The Netherlands; Theoretical Biology & Bioinformatics, Utrecht University, Utrecht 3512 JE, The Netherlands; Laboratorium of Microbiology, Wageningen University & Research, Wageningen 6700 HB, The Netherlands; Life Sciences & Health, Centrum Wiskunde & Informatica, Amsterdam 1098 XG, The Netherlands; Theoretical Biology & Bioinformatics, Utrecht University, Utrecht 3512 JE, The Netherlands; Theoretical Biology & Bioinformatics, Utrecht University, Utrecht 3512 JE, The Netherlands

## Abstract

**Motivation:**

The microbes that live in an environment can be identified from the combined genomic material, also referred to as the metagenome. Sequencing a metagenome can result in large volumes of sequencing reads. A promising approach to reduce the size of metagenomic datasets is by clustering reads into groups based on their overlaps. Clustering reads are valuable to facilitate downstream analyses, including computationally intensive strain-aware assembly. As current read clustering approaches cannot handle the large datasets arising from high-throughput metagenome sequencing, a novel read clustering approach is needed. In this article, we propose OGRE, an Overlap Graph-based Read clustEring procedure for high-throughput sequencing data, with a focus on shotgun metagenomes.

**Results:**

We show that for small datasets OGRE outperforms other read binners in terms of the number of species included in a cluster, also referred to as cluster purity, and the fraction of all reads that is placed in one of the clusters. Furthermore, OGRE is able to process metagenomic datasets that are too large for other read binners into clusters with high cluster purity.

**Conclusion:**

OGRE is the only method that can successfully cluster reads in species-specific clusters for large metagenomic datasets without running into computation time- or memory issues.

**Availabilityand implementation:**

Code is made available on Github (https://github.com/Marleen1/OGRE).

**Supplementary information:**

Supplementary data are available at *Bioinformatics* online.

## 1 Introduction

Metagenomics aims at identifying and characterizing the micro-organisms that live in an environment by analyzing their combined genomic material. Second-generation sequencing allows for sequencing genomic material at a relatively low cost, and results in a dataset that contains large amounts of short reads. Splitting these metagenomic datasets into smaller and more manageable clusters of similar genomes facilitates downstream analyses. For example, several strain aware assemblers work well for small datasets, such as viral quasispecies, but may have difficulties processing metagenome-sized datasets (Baaijens *et al.*, 2017; Baaijens and Schönhuth, 2019; Gregor *et al.*, 2016) that may contain $\geq 10^{7}$ read pairs of 2×150 nt. Clustering reads into smaller groups based on overlaps prior to assembly would scale down the problem: a cluster is much smaller than the full metagenome, while still keeping reads from similar genomes together. Thus, clustering reads based on overlaps is a promising approach towards reconstructing strain-specific genomes (Howe *et al.*, 2014; Tanaseichuk *et al.*, 2012; Wu and Ye, 2011). This article presents a novel overlap graph-based read clustering approach to cluster short reads from metagenomics data at the species level.

Clustering reads into groups that belong to the same species is readily done by mapping the reads to a reference genome, but this is infeasible for organisms without close relatives in the database. Several reference-free read binners for metagenomic short read datasets are available. All of these are based on *k*-mer profiles. Similarities in the coverage and relative frequency of short *k*-mers are used to identify reads that originate from the same genome (Wang *et al.*, 2012; Wu and Ye, 2011). Abundancebin (Wu and Ye, 2011), TOSS (Tanaseichuk *et al.*, 2012) and MBBC (Wang *et al.*, 2015) use this property to derive the species’ abundances and cluster reads accordingly. These methods rely on the assumption that species in the metagenome tend to occur at different abundance levels. MetaCluster 5.0 (Wang *et al.*, 2012) first filters reads from extremely low abundant (≤5×) species based on their *k*-mer frequencies. Next, long *k*-mers (36 nt), which are unique to a genome, are used to cluster reads from high-abundance species (>10×). The remaining reads (from low-abundance species) are clustered based on intermediate length *k*-mer profiles (22 nt).

As demonstrated in our results (Section 3.2), the existing read binning methods are capable of providing a clustering for small datasets (<2.5 million reads). There are currently no methods available that can group commonly sized ($\geq 10^{7}$ read pairs of 2×150 nt) metagenome datasets into taxonomically meaningful clusters.

In this article, we present OGRE, an Overlap Graph-based Read clustEring method. Earlier work (Baaijens *et al.*, 2017) pointed out that overlap graph-based instead of *k*-mer based approaches can have decisive benefits when trying to distinguish between genomes at the strain level. The intuition behind overlap graph-based clustering and assembly is as follows: if one were to construct an overlap graph from reads that stem from a single genome, then the graph consists of a single component when horizontal coverage is complete and vertical coverage over the entire genome is sufficiently high. When an overlap graph contains reads from multiple genomes (i.e. a metagenome) then the components belonging to different genomes will be connected only in regions where two genomes are highly similar. An overlap graph is thus an intuitive tool for metagenomic read clustering, as connected components can be identified with groups of reads that stem from similar genomes.

While the use of overlap graphs is not uncommon for Next-Generation Sequencing (NGS)-based assembly (Baaijens *et al.*, 2017; Simpson and Durbin, 2010), no efficient implementation of an overlap graph-based clustering approach exists in the current literature. In this article, we present OGRE, the first computationally feasible overlap graph-based read clustering approach for high-throughput sequencing data, with a focus on shotgun metagenomes. OGRE constructs an overlap graph where reads are represented as nodes and edges reflect overlaps between reads. The algorithm employs Minimap2 (Li, 2018) to identify overlaps between reads. We make use of machine learning to predict which of these overlaps correspond to reads from the same species. For each step, we develop algorithmic approaches to enable OGRE to handle metagenome-sized read data. Finally we use Mash (Ondov *et al.*, 2016) to combine highly similar clusters. We show that OGRE is capable of clustering large metagenomic datasets into species-specific clusters without running into memory- or time issues. This makes OGRE, to the best of our knowledge, the first method to yield clusters of reads with high species purity from high-diversity shotgun metagenomic datasets.

## 2 Materials and methods 

It is our aim to design a method that clusters metagenomic reads into species-specific clusters by constructing an overlap graph and identifying the connected components within this graph. The algorithm needs to be able to efficiently construct an overlap graph from a large metagenomic dataset typically containing tens of millions of reads, and efficiently identify the clusters in the overlap graph. Here, we first give a global overview of OGRE, further details are discussed in the following sections.

**Fig. 1.**
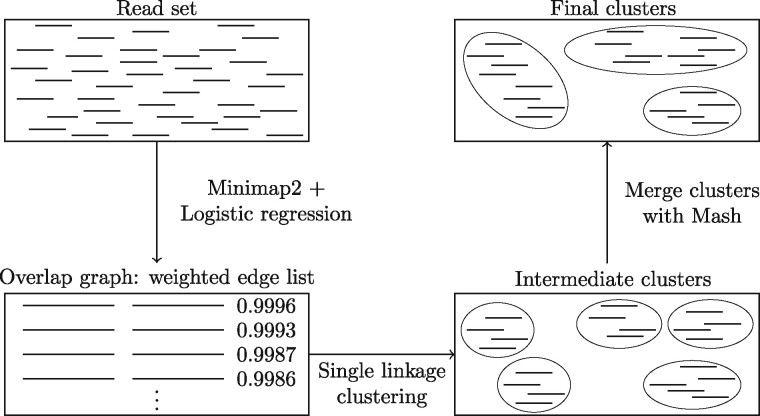
Workflow of OGRE

OGRE consists of four steps: (1) construct an overlap graph, (2) from the list of overlaps select those that are expected to link two reads from the same species, (3) cluster reads that are in the same connected component in the overlap graph and (4) merge highly similar clusters (Fig. 1). In steps (3) and (4), one can impose a maximum cluster size to avoid unrealistically or inconveniently—for downstream analysis—large clusters.

For overlap graph construction, we use Minimap2 (Li, 2018), a tool that uses a heuristic approach to rapidly identify read overlaps. For each overlap, we compute a quality score to obtain an overlap graph with weighted edges, where high weights correspond to overlaps linking reads that are likely to stem from the same species. Weights are in the interval [0,1], and edges (overlaps) with a weight <0.5 are removed from the graph. We then cluster reads such that pairs of reads with a high overlap score are in the same cluster using an efficient parallel implementation of single linkage clustering. We observed that two or three clusters may cover the same part of the genome (see Section 3). Therefore, clusters that show high sequence similarity are merged using Mash (Ondov *et al.*, 2016). These four steps are further described in Sections 2.1, 2.2, 2.3 and 2.4, respectively.

### 2.1 Overlap graph construction

Minimap2 outputs a list of pairs of overlapping reads with information corresponding to this overlap. It offers two possible output formats: a PAF file (Li, 2016) and a SAM file (Li *et al.*, 2009). We experienced issues with the PAF format, as it often reported only part of the overlap between two reads, which could then be mistaken for an overlap in the middle of a read and removed by our post-processing procedure. The SAM format does not suffer from this, as it outputs the CIGAR string from which the complete overlap range can be deduced. After running Minimap2, we discarded overlaps in the middle of reads as these make no sense in terms of clustering or assembly. This leaves us with a list of overlapping pairs of reads, hence a list of overlap graph edges. We chose settings for Minimap2 that allowed for identification of a large number of overlaps with a length of ≥60 bases (*k* = 21, *w* = 11, *s* = 60, *m* = 60, *n* = 2, *r* = 0, *A* = 4, *B* = 2, –end-bonus = 100, see https://lh3.github.io/minimap2/minimap2.html). For an evaluation of several Minimap2 parameter settings, see Supplementary Table S3.

The list of overlaps produced by Minimap2 contains overlaps where both reads originate from the same species as well as pairs where the reads come from different species. We aim to discard the latter by predicting for each overlap whether the reads originate from the same species based on a measure of overlap strength. For this, we compute two metrics that could be relevant: the overlap length and a matching probability based on Phred scores [also used in Baaijens *et al.* (2017)]. The predictive power of these metrics will be assessed in the results (Section 3.4).

The Phred-based overlap score is computed as follows. Suppose we have an overlap for which we observe the two read sequences $s_{1},s_{2}\in {\left\{A,C,G,T\right\}}^{n}$ with corresponding Phred score sequences *p*_1_, *p*_2_. While *s*_1_ and *s*_2_ denote the *observed* read sequences, we denote the *true* (unknown) read sequences by *σ*_1_ and *σ*_2_. The probability that *s*_1_ and *s*_2_ have the same base in the *i*th position can be calculated as follows:

$$\begin{array}{c} P(\sigma_{1,i}=\sigma_{2,i})=\sum_{b=A,C,G,T}P(\sigma_{1,i}=\sigma_{2,i}=b)\\ =\sum_{b=A,C,G,T}P(\sigma_{1,i}=b)P(\sigma_{2,i}=b), \end{array}$$

where

$$P(\sigma_{1,i}=b)=\{\begin{array}{cc} 1-10^{\frac{-p_{1,i}}{10}} & \;\text{if}\;s_{1,i}=b\\ \frac{1}{3}10^{\frac{-p_{1,i}}{10}} & \;\text{otherwise}. \end{array}$$

From this, we compute the Phred-based matching probability, i.e. the probability that two sequences are identical, which is defined as the probability that two bases are identical multiplied over the full overlap:

$$\sqrt[{n}]{\prod_{i=1}^{n}P(\sigma_{1,i}=\sigma_{2,i})}.$$

Using the *n*th root prevents that long overlaps are penalized more heavily than short ones, while longer overlaps of equal quality should be preferred over shorter ones.

Minimap2 produces a SAM file containing a large header and for each overlap the read IDs, CIGAR strings, mapping positions, segment sequences and Phred scores. In our initial tests with a dataset (CAMI_low, see Section 2.5) of approximately 50 million reads, thus approximately 50 000 000^2^ pairs of reads, the output file exceeded 1 TB—once the file reached a size of 1 TB, we stopped the process to prevent storage issues, so we do not know how large the file could get. However, for our purposes, we only need a list of overlaps characterized by the read IDs, the overlap length and the Phred-based overlap quality score, which requires a substantially smaller amount of space. To prevent the production of a large output file we split the set of reads into *k* equal subsets $s_{1},\ldots ,s_{k}$ and ran Minimap2 for every pair of subsets $\left(s_{i},s_{j})\in \{s_{1},\ldots ,s_{k}\}\times \{s_{1},\ldots ,s_{k}\right\}$. Due to its heuristic nature Minimap2 produces a slightly different output for (*s_i_*, *s_j_*) compared to (*s_j_*, *s_i_*), i≠j, that is, it matters which subset is used as a reference and which one as a query. We therefore ran both, resulting in $k^{2}-k$ small output files. Note that when *i *=* j* we only need to run Minimap2 once. For each file, we only kept the read IDs, CIGAR strings, read sequences and Phred scores and removed the remaining redundant information. We computed the overlap length and the Phred-based matching probability from the CIGAR strings, the read sequences and Phred scores, after which the latter three were discarded. This approach is good practice when using parallel resources (Hadoop, 2019).

Next, for each pair of subsets (*s_i_*, *s_j_*), we combined the two overlap files (one obtained with the first subset as reference and the second as query, and one obtained with the reverse) and removed all overlaps that occurred twice. The resulting k+(k−1)+⋯+2+1=k*(k+1)/2 overlap files were merged into a single overlap file that contained a list of overlaps characterized by the corresponding read IDs, overlap length and Phred-based overlap score. This file is the list of overlap graph edges.

### 2.2 Filtering overlaps between reads from different species

We wish to give each edge a weight that indicates the strength of the overlap, and remove overlaps with a low weight as we suspect those to be linking reads from different species. We use logistic regression to determine the edge weights. Logistic regression is a machine learning method that predicts for each sample a binary class based on a sigmoid function applied to a linear transformation of sample characteristics:

$$f(x)=\frac{1}{1+\text{exp}(-\beta_{0}-\sum_{i=1}^{m}\beta_{i}x_{i})},$$

where $x\in R^{m}$ is the vector of sample characteristics. Note that f(x)∈[0,1]. Samples with f(x)>0.5 are predicted to be in one class, the other samples in another. In our case, the classes are ‘same species’ and ‘different species’, where f(x)>0.5 corresponds to ‘same species’, *x* represents an edge, and the characteristics are the overlap length and the Phred-based overlap score. The edge weights in the overlap graph are defined as the output value of the logistic regression *f*(*x*), and edges with f(x)<0.5 are removed. Values for $\beta_{0},\ldots ,\beta_{m}$ are obtained by training the model on past data where the true class of each sample is known. The CAMI database [Critical Assessment of Metagenomic Interpretation, Sczyrba *et al.* (2017)] contains many synthetic datasets that can be used as training data. When applying OGRE to one of the CAMI datasets, we trained the logistic regression model on the other datasets to avoid overlap between training and testing data. Examples of training datasets and resulting regression coefficients are provided in Supplementary Table S4. A ready-to-use training dataset is included on Github, but one could supply their own training data if desired. We further discuss our training approach in Section 3.5.

### 2.3 Implementation of single linkage clustering algorithm

**Fig. 2.**
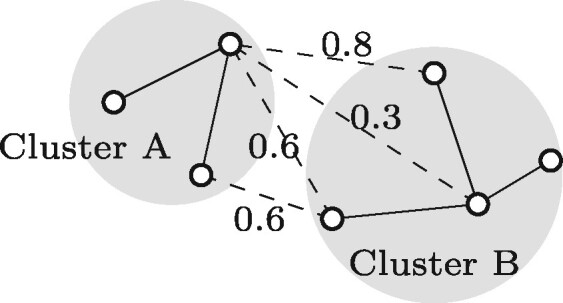
Example of the single linkage clustering. The solid lines denote edges between nodes in the same cluster, the dashed lines denote edges between nodes from different clusters with their overlap scores. The overlap score between clusters A and B is the maximum overlap score over the four connections between the clusters, which equals 0.8

Single linkage clustering was selected as it was the most efficient clustering algorithm. The list of overlaps—constructed as described above—describes the overlap graph and forms the starting point of the clustering algorithm. Initially each read forms a cluster by itself, and every iteration the single linkage algorithm merges the two clusters with the highest overlap score between them. The overlap score between clusters *A* and *B* is defined as the maximum overlap score over all pairs of read ends $r=(r_{1},r_{2}),\;r_{1}\in A,\;r_{2}\in B$ (Fig. 2). Sorting the overlaps by decreasing overlap score provides the order in which clusters are merged. After sorting, the merging algorithm’s complexity is linear in the number of edges: for each line in the file, the two clusters that contain the reads of that line are merged.

While looking for the connected components in a graph all edges are used, so the order in which clusters are merged may seem irrelevant. However, one may wish to limit the cluster size. The cluster size threshold depends on why a user performs the read clustering in the first place. For example, if the end goal is strain-aware assembly, then the maximum cluster size depends on the assembler’s capacity. An additional advantage is that connected components may contain multiple species, so limiting the cluster size improves cluster purity, see Section 3.5.

The above algorithm has a complexity of O(n log n+n), namely the complexity of the merge sort algorithm used by the bash command ‘sort’ plus the complexity of the single linkage algorithm, where *n* is the number of edges in the graph or overlaps. Note that *n* is in the order of 10^9^ (Supplementary Table S6). This makes executing the algorithm non-trivial: any serial clustering algorithm has to go through all the edges in the overlap list and will thus take an infeasible amount of time. We therefore use several techniques to speed up the method.

**Fig. 3.**
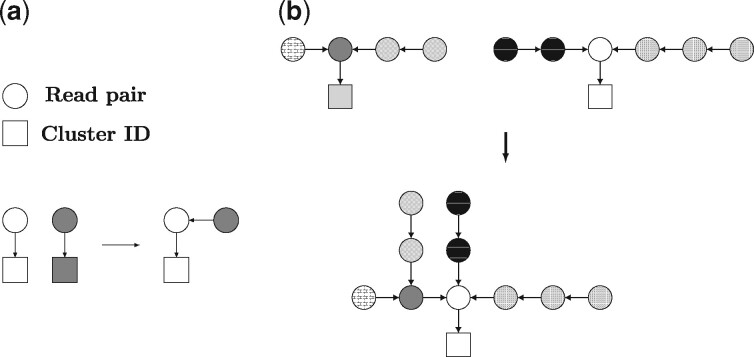
Merging two clusters of (**a**) a single node each and (**b**) multiple nodes by changing the value of one node to be equal to the ID of the other node. An arrow points from a key to its value in the hash table. An arrow from a circle to a square indicates that this read is in the cluster corresponding to the square. An arrow from one circle to another indicates that the read from which the arrow departs is in the same cluster as the read that the arrow points to. Fill patterns allow for identification of nodes between the two steps

*Hash table* We store our clustering in a hash table where each key is a node (read ID) and its value is the ID of the cluster the read belongs to (see left part of Fig. 3a). Recall that initially each read forms a cluster on its own, and in each iteration, two clusters are merged. Merging two (single-node) clusters is done by updating the value of one node to be equal to the read ID of the other node instead of the cluster ID. This results in a cluster with two nodes where the value of one node is the read ID of the second node, and the value of the second node is the cluster ID (Fig. 3a). When merging two clusters that contain multiple nodes we update only the value of the node that points to the cluster ID, referred to as cluster head, in one of the clusters to be equal to the head of the other cluster (Fig. 3b). As a result, only a single value needs to be updated when two (potentially large) clusters are merged. See below for details on obtaining the cluster head.

*Updating the cluster with the shorter maximum chain* Suppose we have arrived at an iteration where the algorithm considers the overlap between reads *r*_1_ and *r*_2_. Then the clusters containing reads *r*_1_ and *r*_2_, denoted by *C*_1_ and *C*_2_, respectively, need to be merged. We need to identify the heads of *C*_1_ and *C*_2_ by traversing the path through the hash table from reads *r*_1_ and *r*_2_ to the respective cluster heads. The number of operations for this procedure equals the length of the path from *r*_1_ to the head of cluster *C*_1_ plus the length of the path from *r*_2_ to the head of cluster *C*_2_. For example, consider the cluster in the lower part of Figure 3b, and suppose the right-most node in this cluster is *r*_1_. To find the cluster ID (the square), one needs to traverse three nodes, hence four operations are required. We thus need to keep the length of the longest chain in each cluster short. When merging two clusters, we therefore redirect the head of the cluster with the shorter maximum chain within the cluster to the head of the other cluster. The length of the maximum chain for each cluster is stored in a dictionary. As a result, the maximum chain within a cluster of size *m* will never exceed 1+⌊m/2⌋, see Appendix D for a proof. This leads to an overall complexity of the single linkage clustering algorithm of O(n log n+nm+n).

**Fig. 4.**
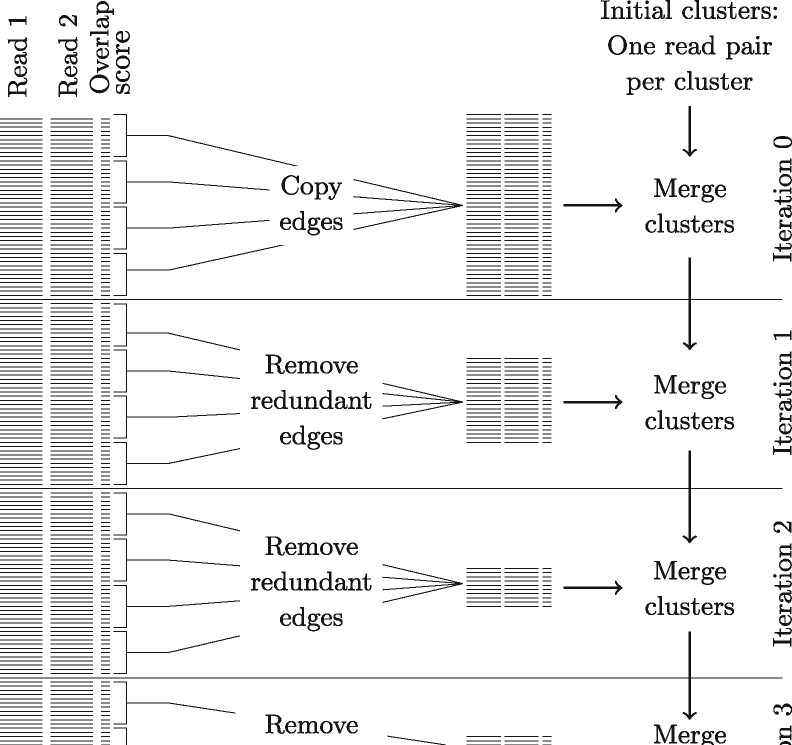
Schematic overview of the parallellization approach. In each iteration, each parallel process receives a set of edges (square brackets). It removes redundant edges and passes the remaining edges on to the main process, which then merges clusters

*Parallelization of the single linkage clustering algorithm* Although the single linkage clustering algorithm, an inherently sequential algorithm, is very fast and simple, the overlap file contains from tens of millions up to billions of overlaps and going through these one by one could take up to months for a metagenomic dataset. After going through a number of these lines, hence after several clusters have been merged, many overlaps become redundant: they are overlaps between pairs of reads that are in the same cluster already, or merging their clusters yields a too large new cluster. We prefilter these redundant edges using a parallel process, see Figure 4. First, the overlap file is split up in batches of a predetermined number of overlaps, and the batches are processed iteratively. At iteration *i*, batch *i* is split into minibatches from which redundant edges are removed. To determine whether an overlap is redundant, the algorithm checks whether the two reads are in the same cluster already which has complexity O(n log n+nm+n), see the previous paragraph. If the reads are in different clusters the algorithm checks whether the total size of the two clusters does not exceed the maximum allowed cluster size, which can be done efficiently since the algorithm keeps track of all cluster sizes using a hash table. The minibatches are processed in parallel and the remaining edges are returned to the main process. Starting from the clustering obtained in iteration *i* – 1, the main process then continues merging clusters based on this reduced list of edges. The resulting clustering forms the starting point for the next iteration. After several iterations more than 99.9% of the edges have become redundant, so the main process has only few edges left for merging clusters which largely speeds up the process.

### 2.4 Merging clusters with Mash

Steps (1)–(3) may result in parts of the genome of one species being covered by reads that are in two or more different clusters (see Section 3.6). Such clusters yield a reduced read coverage which is undesirable for strain-aware assembly. Therefore, we use Mash (Ondov *et al.*, 2016) to combine read clusters based on sequence similarity. In short, Mash utilizes MinHash sketches to rapidly estimate the Mash distance between large sequences, sequence sets or read sets. The Mash distance is highly correlated with alignment-based metrics such as the average nucleotide identity (Ondov *et al.*, 2016). We applied Mash to compute the distance between any two read clusters as well as the *P*-value to estimate the confidence of a given distance, corrected for multiple testing using a Benjamini–Hochberg correction. Two clusters were merged when both the adjusted *P*-value and the distance were small.

### 2.5 Data

We tested OGRE on datasets provided by the first CAMI challenge as well as a subset from the mousegut dataset from the second CAMI challenge (Sczyrba *et al.*, 2017), see Supplementary Table S1. For the mousegut data, we selected five samples (S5, S31, S33, S54, and S57) such that for several species more than 10 strains were included. All datasets contain simulated short Illumina reads from a mixture of strains that can be grouped into species. The datasets differ in complexity: they contain between ≈50 million (CAMI_low) up to ≈77 million (toy_medium) read pairs, 27 (CAMI_low) up to 405 (CAMI_mousegut) species and 30 (toy_low) upto to 1074 (CAMI_high) strains. Reads were quality trimmed using cutadapt (Martin, 2011) with a quality cutoff of 30, and reads shorter than 80 nt (after quality trimming) were removed.

## 3 Results

This section is organized as follows. In Sections 3.1 and 3.2, the performance of OGRE is compared to other read binners for small and medium datasets, respectively. Section 3.3 presents runtime and memory usage, and Sections 3.4–3.6 discuss the performance of steps 2–4 of OGRE individually. All experiments are run on a system with 24 CPUs and 128 GB of RAM.

### 3.1 Only OGRE and Abundancebin can successfully cluster datasets with 5–10 million paired-end reads

We first tested the performance of Abundancebin (Wu and Ye, 2011), MetaCluster 5.0 (Wang *et al.*, 2012), MBBC (Wang *et al.*, 2015)—three state-of-the-art reference-free clustering methods—and OGRE on small datasets containing 5–10 million paired-end reads of 2–4 species. These datasets were obtained by including only the reads that belong to a chosen subset of the species in the CAMI_low dataset. We created three datasets with varying abundance distributions and number of species, see Supplementary Table S2. Results are provided in Supplementary Table S5. OGRE was run without Mash and unlimited cluster size. We could not run OGRE on datasets provided in the papers of the other tools and directly compare our results, as for these datasets, only fasta files are available while OGRE uses the additional information contained in the Phred scores.

For the dataset with the smallest number of reads (2species_a), MBBC did not complete the read clustering within 2 months, so we terminated it. We therefore did not attempt to run MBBC on larger datasets.

MetaCluster 5.0 gave clusters containing reads from a single species. However, these clusters were very small: < 1% of the reads were clustered. This may be because MetaCluster 5.0 can only process data where all reads have the same length, which forced us to truncate the reads at the length of the shortest read after QC (80 nt).

Abundancebin allows the user to predefine the number of clusters. When Abundancebin was provided with the correct number of clusters, it could not cluster subset 2species_b. For both 2species_a and 4species, when Abundancebin was instructed to find two or four clusters, those clusters contained reads from multiple species. Abundancebin clustered all reads. Not providing the number of clusters to Abundancebin resulted in a single cluster with all reads for subset 2species_a, and did not finish within 2 months for the other datasets.

OGRE found clusters for all datasets. Note that OGRE does not require the number of clusters as an input. For all datasets OGRE provided single-species clusters, and the number of clusters per species varied from 1 to 333. For species with high read coverage >98% of all reads were placed in a cluster, whereas for low-coverage species only 3% of the reads were clustered, which is inevitable as low coverage implies few overlaps, if any.

In summary, for the datasets where Abundancebin found clusters it was able to cluster all reads but could not achieve single-species clusters. MetaCluster 5.0 obtained pure clusters, but only less than 1% of reads were clustered. OGRE provided pure clusters and clusters more than 98% of the reads for species with high read coverage (>10×).

### 3.2 Clustering CAMI_low with available read clustering methods gives memory and time issues

We have attempted to perform read clustering of the CAMI_low dataset (Supplementary Table S1) using Abundancebin (Wu and Ye, 2011), MetaCluster 5.0 (Wang *et al.*, 2012) and MBBC (Wang *et al.*, 2015). These methods were unable to finish within reasonable time when applied to subsets of the data. MBBC spent over a month on a subset containing 5% of the species before we stopped the process. Abundancebin was able to handle this subset, but after running it on 20% of the reads for 3 weeks, we stopped the process. MetaCluster 5.0 could manage 20% of the data, again clustering 1% of the reads, but was not able to handle the full dataset. Table 1 shows for each method which datasets it can handle.

**Table 1.** An overview of four clustering methods, showing whether these methods are able to cluster the datasets considered in this article within a month on a 24 CPU system

Dataset	MBBC	Abundancebin	MetaCluster 5.0	OGRE
2species_a	No	Yes	Yes	Yes
2species_b	No	No	Yes	Yes
4species	No	Yes	Yes	Yes
CAMI_low—5%^a^	No	Yes	Yes	Yes
CAMI_low—20%^a^	No	No	Yes	Yes
CAMI_low	No	No	No	Yes
CAMI_medium	No	No	No	Yes
CAMI_high	No	No	No	Yes
toy_low	No	No	No	No
toy_medium	No	No	No	Yes
toy_high	No	No	No	Yes

a 5 and 20% of the species were randomly selected and included in the subset.

### 3.3 No memory- and runtime issues with OGRE for all but one dataset

Runtimes for OGRE are presented in Supplementary Table S6, where we ran steps 1–3 of OGRE and no limit on the cluster size. Note that the toy_low dataset is missing: our method could not be run on this dataset within reasonable time. This was due to the large number of overlaps that Minimap2 identified, resulting in a prohibitively large runtime and overlap file. For the remaining five datasets our parallelization approach made overlap graph-based clustering feasible on a multicore system. The overall procedure took between 60 and 160 h on a 24 CPU system.

The main issue with constructing an overlap graph by directly running Minimap2 on the CAMI data is the excessively large output file (over 1 TB). The algorithm described in Section 2.1 was developed to overcome this issue, hence the main performance indicator for this step was the size of the resulting overlap files. The final overlap file had an acceptable size (up to 360 GB, see Supplementary Table S6).

### 3.4 The prediction step removes many different species overlaps and keeps most same species overlaps

For a given overlap, we aim to predict whether the corresponding reads originate from the same genome based on the overlap length and a Phred-based matching probability.

First, we compared the distributions of the overlap length and the Phred-based matching probability for same-species versus different-species pairs of reads. We randomly selected overlaps from each of the datasets: 1 000 000 same-species pairs and 1 000 000 different-species pairs. Comparing the distributions for overlap length and Phred-based matching probability for same-species overlaps and different-species overlaps (Supplementary Fig. S1) shows that the Phred-based probability score can be a valuable predictor for whether an overlap corresponds to reads from the same species, while the overlap length seems less indicative.

Supplementary Table S7 shows train and test accuracies for the five datasets from the first CAMI challenge. The first column indicates the dataset for which the reads are to be clustered (the test data). The training data were constructed by randomly selecting 10 000 overlaps between reads from the same species and 10 000 overlaps between reads from different species from each of the four datasets other than the test data, and combining those into a single training dataset. Note that there was a big gap between the training and the test accuracy, which is due to a combination of two factors. The test data (containing the complete overlap graph) were highly unbalanced: over 99% of the edges corresponded to an overlap between reads from the same species. This, combined with the model being far better at recognizing same species overlaps than different species overlaps, yielded a higher accuracy for the test data than for the training data.

This step aims to discard as many overlaps between reads from different species as possible—such overlaps lead to merging clusters with reads from different species—while keeping most of the same-species overlaps. We consider the fraction of same species- and different species overlaps that were discarded by the logistic regression classifier. Supplementary Table S7 shows that while for most datasets more than 90% of the same species overlaps were kept, around half of the overlaps that link different species were discarded, and for CAMI_low this was even over 90%.

### 3.5 Clustering obtained after step 3 gives sensible results for all datasets but one

For each dataset the clustering was performed with a maximum cluster size of 3300, 17 000 and 33 000 reads (corresponding to at most 1 million, 5 million and 10 million base pairs, respectively), as well as with unlimited cluster size (i.e. with all overlaps included). All clusters of <20 reads were discarded, and these reads were considered as not clustered. We first evaluated the clustering obtained after step 3 (Fig. 1). The effects of applying Mash, step 4, will be presented in Section 3.6.

In a perfect clustering each read is clustered, all reads from the same species are in one cluster and each cluster contains reads from only one species. We thus assessed the fraction of reads that was clustered, the number of clusters per species and the number of species per cluster. The fraction of reads clustered was generally high for species with high read coverage (>10×, Supplementary Fig. S2). As maximum cluster size increased, the number of clustered reads increased as well. For toy_high only 0.01% of the reads were clustered regardless of the maximum cluster size (Supplementary Fig. S2e). The reads from species with a low read coverage were spread over many clusters, while reads from a high coverage species were placed in a single cluster (Supplementary Fig. S3). The maximum number of species in a cluster increased with maximum cluster size, while the total number of clusters decreased (Supplementary Fig. S4).

**Fig. 5.**
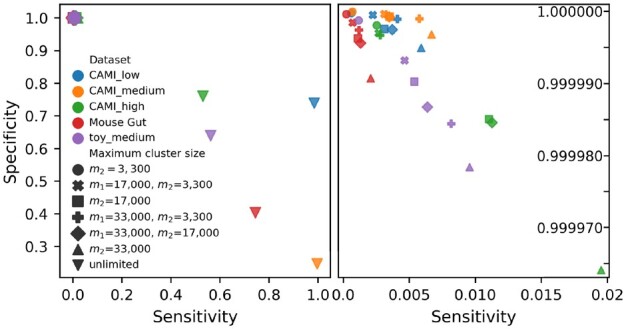
Sensitivity (fraction of pairs of reads from the same species that are in the same cluster) and specificity (fraction of pairs of reads from different species that are in different clusters) obtained with various settings of OGRE. Two reads in the same cluster that stem from the same (different) species are a true (false) positive, while two reads in different clusters from the same (different) species are a false (true) negative. The complete figure is shown on the left. *m*_1_ is the maximum allowed clustering size after step 4, *m*_2_ is the maximum allowed cluster size after step 3. If *m*_1_ is not given the clustering was obtained without step 4. The figure on the right zooms in on the region in the top left corner of the full figure and does not show the results obtained with unlimited cluster size.

**Fig. 6.**
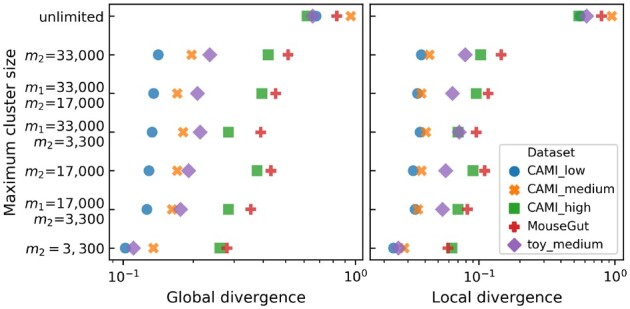
Maximum divergence between two genomes in the same cluster, averaged over all clusters. *m*_1_ is the maximum allowed clustering size after step 4, *m*_2_ is the maximum allowed cluster size after step 3. If *m*_1_ is not given the clustering was obtained without step 4. Global divergence is the fraction of the bases that is not matched between two reads. For local divergence only the overlapping regions of two reads are considered

To gain insight in the trade-off between the number of species per cluster and the number of clusters per species we looked at sensitivity and specificity. Two reads in the same cluster that stem from the same (different) species are a true (false) positive, while two reads in different clusters from the same (different) species are a false (true) negative. Figure 5 shows that when the maximum cluster size increased, sensitivity increased at the cost of specificity. These differences were major when comparing unlimited cluster size to limited cluster size, while the differences among the clusterings with limited cluster size were minor. The low sensitivity indicates that many pairs of reads from the same species were in different clusters, and thus many clusters per species existed. Figure 6 shows the maximum divergence between any two genomes in a cluster, averaged over the clusters. Here, global divergence is the divergence over the full genome, while local divergence solely considers the part where two genomes overlap. Naturally divergence increased with cluster size.

### 3.6 Merging clusters with Mash improves the clustering

We evaluated the effect of using Mash (step 4, see Fig. 1), to obtain clusters of size at most *m*_1_, and compared it to a clustering obtained with steps 1–3 where cluster sizes after step 3 (single linkage clustering, see Fig. 1) were restricted to be at most *m*_2_ = *m*_1_. Note that when Mash produces clusters of size at most *m*_1_, step 3 should give clusters of size at most $m_{2}<m_{1}$. Mash used under 12 h on 12 CPUs.

Figure 5 shows no decisive benefit from using Mash as a fourth step over clustering with steps 1–3 only, it merely leads to a different trade-off between specificity and sensitivity. This is supported by Supplementary Figures S3 and S4, which show that applying Mash in step 4 reduced the number of clusters but increased the number of species per cluster. However, Mash had clear benefits when looking at within-cluster genome divergence: a clustering of maximum size 17 000 or 33 000 obtained with Mash had a lower divergence than clusters with the same maximum size obtained with steps 1–3 only (Fig. 6).

**Fig. 7.**
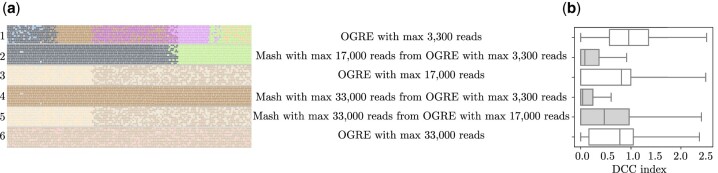
A comparison of six clustering strategies for CAMI_low. (**a**) A fraction of the source genome with reads mapped to their source position viewed through IGV (Robinson *et al.*, 2011). The colors of the reads indicate the cluster they belong to for six different clustering strategies. (**b**) The distribution of the Diversity of Covered Clusters (DCC) index over the three million randomly selected positions on the source genome for six clustering strategies

Figure 7a shows a part of the source genome of one strain with reads that are mapped to their original (known) positions. Reads with the same color belong to the same cluster. Rows 1, 3 and 6 in Figure 7a show that step 3 often resulted in two clusters covering the same part of the genome, while Mash often merged these clusters into one (rows 2 and 4). For $m_{1}=33\;000$ and $m_{2}=17\;000$ (row 5) Mash did not merge the two visible clusters, since these two clusters each contained 17 000 reads.

To obtain an aggregate overview of the clustering performance of Mash based on the number of clusters that cover a position on the source genome, we introduce the Diversity of Covered Clusters (DCC):

$$DCC_{k}=-\sum_{i=1}^{c}p_{i}\;\text{log}(p_{i}),$$

with *k* a coordinate on the source genome, *c* the number of clusters covering position *k* and *p_i_* the proportion of reads covering position *k* that belong to cluster *i* as a fraction of the total number of reads covering position *k*. A low *DCC* indicates that few clusters cover a base position, and ideally *DCC* = 1. We calculated *DCC_k_* for three million randomly chosen base positions on the source genomes of CAMI_low. The distribution of *DCC_k_* (Fig. 7b) shows that Mash lowered the DCC index significantly, and that the best results were obtained when running OGRE with a maximum cluster size of 3300 after step 3 and 33 000 after step 4.

### 3.7 Multispecies clusters correspond to conserved genomic regions

Several clusters contained multiple species. For datasets CAMI_low, CAMI_medium and CAMI_high, many of the reads in clusters that contained at least two species map to protein-coding genes (Supplementary Fig. S5). Zooming in on some of the clusters with two species showed that the regions linking the different genomes corresponded to genes encoding highly conserved protein products, including DNA-directed RNA polymerase subunit beta. This indicates that the overlap graph contained hubs that correspond to shared sequences in the genome, explaining why an overlap graph-based read clustering approach yields some multispecies clusters.

### 3.8 Weak clustering for toy_high inherent to the data

The toy_high dataset is the only dataset for which a weak clustering was obtained. This is due to the low read coverage per strain: 47% of the strains has a read coverage below 2×, and 75% of the strains has a read coverage below 5×. This, in combination with the short read length of the toy datasets, makes both clustering and assembly nearly impossible.

### 3.9 Testing the use of OGRE to enhance assembly

As described before, there is substantial motivation for clustering reads before assembly to enhance metagenome assembly at strain-level resolution (Howe *et al.*, 2014; Tanaseichuk *et al.*, 2012; Wang *et al.*, 2012). We conducted a preliminary test, while leaving the development of a complete clustering and assembly pipeline for future work. We assembled reads in three steps: (1) cluster reads using OGRE, (2) assemble reads of each cluster separately using SPAdes (Bankevich *et al.*, 2012) and (3) assemble the *full* dataset of unassembled reads with SPAdes using the contigs obtained in step (2) as a guidance with the SPAdes option-trusted contigs. Note that step (3) is necessary as for some species reads are present in different clusters. We used an OGRE clustering with $m_{1}=33\;000,\;m_{2}=3300$, as these settings led to the best DCC (Fig. 7) and performed well in terms of divergence (Fig. 6). The assembly was compared with directly assembling the full read dataset and assembly based on a random clustering. MetaQUAST was used to assess the quality of the assembly (Mikheenko *et al.*, 2016). We did not use MetaSPAdes, as earlier work shows that it is outperformed by SPAdes even for metagenome data (Baaijens *et al.*, 2017).

Supplementary Table S8 shows that OGRE improved the contig length (N50 and NA50) quite substantially, and reduced the number of partially unaligned contigs (except for CAMI_medium) and the number of misassemblies compared to an assembly of an equal number of randomly selected reads, and of all reads. Both a random clustering and clustering with OGRE led to an improvement in the recovered fraction of the genome compared to assembly of all reads (Supplementary Table S8).

## 4 Discussion

This article presents OGRE, an overlap graph-based read clustering approach for clustering the reads in large metagenomic datasets. It (1) constructs an overlap graph using Minimap2, (2) filters out a large fraction of the overlaps between reads from different species, (3) clusters reads using single linkage clustering and (4) merges highly similar clusters using Mash. Even though these four key processes of OGRE all have low computational complexity, we encountered computational difficulties due to the size of metagenomic datasets that were resolved within OGRE. First, Minimap2 resulted in an unacceptably large overlap file for our test cases. OGRE therefore applies Minimap2 to parts of the data, removes redundant information from the separate overlap files and merges the resulting files. Second, while the single linkage algorithm is a highly efficient approach for clustering reads from the overlap graph, it is sequential in nature and applying it to the long edge list resulted in unacceptable computation times. As 99% of these overlaps were redundant, we developed a preprocessing approach that filters redundant reads from subsets of the edge lists in parallel and as such made clustering computationally feasible.

Only OGRE and Abundancebin were able to cluster small datasets. While Abundancebin was able to cluster more reads than OGRE, its clusters contained a mix of species as opposed to the single-species clusters provided by OGRE. Abundancebin could only obtain results when the number of clusters is given *a priori*. OGRE was the only clustering approach that could handle metagenome-sized datasets.

For the toy_high dataset insufficient overlaps were found due to the low coverage of the genomes in the dataset. Conversely, OGRE was unable to cluster toy_low due to the long list of overlaps identified by Minimap2. One can set Minimap2 parameters such that it stores fewer overlaps, see Supplementary Table S3.

OGRE allows for limiting the cluster size after both steps 3 and 4. Several configurations were tested, where each provides a different trade-off between sensitivity and specificity. Mash was able to reduce both local divergence and DCC, which reflects the number of clusters that cover a position on the source genome. For all datasets, the best local divergence and DCC were obtained with a maximum cluster size of 3300 after step 3 and 33 000 after step 4.

The logistic regression uses a cutoff for edge removal. This cutoff represents a trade-off between the number of clusters and cluster purity. A low cutoff means that more edges are kept and clusters are merged further, leading to fewer and larger clusters per species and more species per clusters. Conversely, a higher cutoff leads to more and smaller clusters and fewer species per cluster.

In practice, there are two challenges with the concept of overlap graph-based read clustering. First, a connected component in the overlap graph may contain reads from multiple genomes that share part of their sequence. We indeed observed some clusters that contain reads from multiple species. The low local divergence between genomes within a cluster indicates that this corresponded to identical subsequences in genomes, leading to hubs in the overlap graph. This is a common feature in metagenomics data (Koren *et al.*, 2011). A possible future avenue for resolving this could include removing hubs from the network and adding the associated reads to multiple remaining clusters. Second, reads from a single genome may cluster into multiple separate connected components in the overlap graph when read coverage is low. This is consistent with our observations: while the clustering approach worked well for species with a relatively high read coverage, many reads from species with low read coverage (below ∼15×) remained unclustered. Applying a *k*-mer-based read clustering method to the remaining reads may resolve this issue. This is left for future research.

A species-specific read clustering approach for metagenomics such as OGRE may contribute to metagenome assembly by breaking down the complexity of the dataset (Namiki *et al.*, 2012). Combining OGRE with strain-aware assemblers will be valuable for strain tracking in metagenomic datasets. Specifically, we expect that strains that occur in the datasets with a genomic read coverage of >10× will allow species-specific read clusters to be generated that represent >99% of the genome. These smaller read clusters may then be assembled with strain-aware assemblers such as Snowball (Gregor *et al.*, 2016) or Polyte (Baaijens and Schönhuth, 2019).

## 5 Conclusion

This article presents OGRE, an overlap graph-based read clustering approach. We developed a parallelized approach such that an overlap graph-based method becomes feasible even for realistic large metagenomic datasets. This makes OGRE the only direct read clustering method that can handle large datasets.

## Funding

M.B. and A.S. were supported by the Netherlands Organization for Scientific Research (NWO) Vidi grant 639.072.309. M.B. and B.E.D. were supported by NWO Vidi grant 864.14.004. XL was supported by the Chinese Scholarship Council. 

*Conflict of Interest*: none declared.

## Supplementary Material

btaa760_Supplementary_Data

## References

[btaa760-B1] Baaijens J. et al (2017) De novo assembly of viral quasispecies using overlap graphs. Genome Res., 27, 835–848. 28396522 10.1101/gr.215038.116 PMC5411778

[btaa760-B2] Baaijens J.A. , Schönhuth A. (2019) Overlap graph-based generation of haplotigs for diploids and polyploids. Bioinformatics, 35, 4281–4289. 30994902 10.1093/bioinformatics/btz255

[btaa760-B3] Bankevich A. et al (2012) Spades: a new genome assembly algorithm and its applications to single-cell sequencing. J. Comput. Biol., 19, 455–477. 22506599 10.1089/cmb.2012.0021 PMC3342519

[btaa760-B4] Gregor I. et al (2016) Snowball: strain aware gene assembly of metagenomes. Bioinformatics, 32, i649–i657. 27587685 10.1093/bioinformatics/btw426

[btaa760-B5] Apache Hadoop (2019) MapReduce Tutorial. Apache software foundation.

[btaa760-B6] Howe A.C. et al (2014) Tackling soil diversity with the assembly of large, complex metagenomes. Proc. Natl. Acad. Sci. USA, 111, 4904–4909. 24632729 10.1073/pnas.1402564111 PMC3977251

[btaa760-B7] Koren S. et al (2011) Bambus 2: scaffolding metagenomes. Bioinformatics, 27, 2964–2971. 21926123 10.1093/bioinformatics/btr520 PMC3198580

[btaa760-B8] Li H. (2016) Minimap and miniasm: fast mapping and de novo assembly for noisy long sequences. Bioinformatics, 32, 2103–2110. 27153593 10.1093/bioinformatics/btw152 PMC4937194

[btaa760-B9] Li H. (2018) Minimap2: pairwise alignment for nucleotide sequences. Bioinformatics, 34, 3094–3100. 29750242 10.1093/bioinformatics/bty191 PMC6137996

[btaa760-B10] Li H. et al; 1000 Genome Project Data Processing Subgroup. (2009) The sequence alignment/map format and SAMtools. Bioinformatics, 25, 2078–2079. 19505943 10.1093/bioinformatics/btp352 PMC2723002

[btaa760-B11] Martin M. (2011) Cutadapt removes adapter sequences from high-throughput sequencing reads. EMBnet. J., 17, 10.

[btaa760-B12] Mikheenko A. et al (2016) Metaquast: evaluation of metagenome assemblies. Bioinformatics, 32, 1088–1090. 26614127 10.1093/bioinformatics/btv697

[btaa760-B13] Namiki T. et al (2012) Metavelvet: an extension of velvet assembler to de novo metagenome assembly from short sequence reads. Nucleic Acids Res., 40, e155–e155. 22821567 10.1093/nar/gks678 PMC3488206

[btaa760-B14] Ondov B.D. et al (2016) Mash: fast genome and metagenome distance estimation using MinHash. Genome Biol., 17, 132. 27323842 10.1186/s13059-016-0997-x PMC4915045

[btaa760-B15] Robinson J.T. et al (2011) Integrative genomics viewer. Nat. Biotechnol., 29, 24–26. 21221095 10.1038/nbt.1754 PMC3346182

[btaa760-B16] Sczyrba A. et al (2017) Critical assessment of metagenome interpretation-a benchmark of metagenomics software. Nat. Methods, 14, 1063–1071. 28967888 10.1038/nmeth.4458 PMC5903868

[btaa760-B17] Simpson J.T. , Durbin R. (2010) Efficient construction of an assembly string graph using the FM-index. Bioinformatics, 26, i367–i373. 20529929 10.1093/bioinformatics/btq217 PMC2881401

[btaa760-B18] Tanaseichuk O. et al (2012) A probabilistic approach to accurate abundance-based binning of metagenomic reads. In: International Workshop on Algorithms in Bioinformatics. Springer, Berlin, Heidelberg, pp. 404–416.

[btaa760-B19] Wang Y. et al (2012) Metacluster 5.0: a two-round binning approach for metagenomic data for low-abundance species in a noisy sample. Bioinformatics, 28, i356–i362. 22962452 10.1093/bioinformatics/bts397 PMC3436824

[btaa760-B20] Wang Y. et al (2015) Mbbc: an efficient approach for metagenomic binning based on clustering. BMC Bioinformatics, 16, 36. 25652152 10.1186/s12859-015-0473-8 PMC4339733

[btaa760-B21] Wu Y. , Ye Y. (2011) A novel abundance-based algorithm for binning metagenomic sequences using l-tuples. * Journal of Computational Biology, 18,* 535–549. 10.1089/cmb.2010.0245 PMC3123841 21385052

