# Supplementary material for: OGRE: Overlap Graph-based metagenomic Read clustEring
Source: Bioinformatics. 2020 Sep 1;37(7):905–12. doi: 10.1093/bioinformatics/btaa760 (PMC8128468; doi:10.1093/bioinformatics/btaa760)
Supplement: btaa760_Supplementary_Data [file btaa760_supplementary_data.pdf]

Appendix A: Characteristics of datasets

Table S1. Characteristics of seven CAMI datasets. *Four out of the six CAMI datasets contain multiple samples. For these we only used samples S001. **This is a relative complexity as indicated by **?**, see their paper for further details.

Dataset	Sample*	No. reads	Read length (nt)	No. species	No. strains	Complexity**
CAMI mousegut	5, 31, 33, 54, 57	80,080,460	2×150	405	544	-
CAMI_low	-	49,898,179	2×150	27	60	Low
CAMI_medium	S001	66,489,042	2×150	91	232	Medium
CAMI_high	S001	49,901,367	2×150	376	1074	High
toy_low	-	72,855,674	2×100	30	30	Low
toy_medium	S001	77,155,802	2×100	199	225	Medium
toy_high	S001	74,016,648	2×100	375	450	High

Table S2. Characteristics of three small subsets of CAMI_low.

Subset ID	Species included (CAMI OTU)	Coverage per species	No. strains per species	No. read pairs per species
2species_a	220	3	1	41,609
	294	127	2	6,146,574
2species_b	294	127	2	6,146,574
	340	487	1	5,653,696
4species	126	22	1	191,369
	220	3	1	41,609
	223	98	3	1,346,963
	340	487	1	5,653,696

Appendix B: Minimap2 parameter settings

Table S3. Clustering performance for various choices of parameters for Minimap2. The "base" case represents the set of parameters we chose, namely $k = 21$, $w = 11$, $s = 60$, $m = 60$, $n = 2$, $r = 0$, $A = 4$, $B = 2$. As can be seen from the table, the choice of parameters determines the trade-off between having more reads clustered versus the cost of high runtime and memory usage on disk. sp. = species.

Evaluation metric	Base	$k = 17$, $w = 9$	$k = 25$ $w = 15$	$s = 40$	$s = 80$	$m = 80$	$m = 40$	$n = 1$	$n = 4$	$r = 2$	$A = 6$	$A = 2$	$B = 1$	$B = 4$
% reads clustered for sp. 126	0.997	0.999	0.818	0.831	0.997	0.959	1.000	0.997	0.997	0.997	0.997	0.997	0.831	0.997
% reads clustered for sp. 220	0.031	0.066	0.005	0.026	0.031	0.001	0.125	0.031	0.029	0.031	0.030	0.033	0.026	0.032
% reads clustered for sp. 223	0.999	1.000	0.838	0.833	0.999	0.988	1.000	0.999	0.999	0.999	0.999	0.999	0.833	0.999
% reads clustered for sp. 340	1.000	1.000	0.831	0.833	1.000	0.993	1.000	1.000	1.000	1.000	1.000	1.000	0.833	1.000
No. clusters with sp. 126	25	22	35	25	25	178	11	25	25	25	25	25	25	25
No. clusters with sp. 220	55	111	13	55	55	1	204	55	51	53	52	58	55	56
No. clusters with sp. 223	2	2	3	2	2	6	2	2	2	3	2	2	2	2
No. clusters with sp. 340	1	1	1	1	1	6	1	1	1	1	1	1	1	1
Mean no. sp. per cluster [range]	1 [1,1]	1 [1,1]	1 [1,1]	1 [1,1]	1 [1,1]	1 [1,1]	1 [1,4]	1 [1,1]	1 [1,1]	1 [1,1]	1 [1,1]	1 [1,1]	1 [1,1]	1 [1,1]
Total number of clusters	83	136	52	83	83	191	218	83	79	82	80	86	83	84
Size of overlap file	58G	77G	39G	58G	58G	25G	110G	58G	58G	58G	58G	58G	59G	57G
Runtime (mins)	961	1256	439	656	951	430	1652	936	936	936	938	927	670	923

Appendix C: Coefficients obtained with logistic regression

Table S4. Logistic regression coefficients obtained with five training datasets. In each training set we included 10,000 same-species overlaps and 10,000 different-species overlaps from each of the datasets indicated in the first column. When applying OGRE to one of the datasets from the first CAMI challenge we used the training data that does not include the dataset that is to be clustered. When clustering the CAMI mousegut dataset we used the training dataset containing overlaps from CAMI_medium, CAMI_high, toy_medium and toy_high. We excluded the CAMI_low data since this dataset contains very few species with multiple strains.

Training data obtained from	Coefficient for	
	Overlap length	Phred score
CAMI_medium, CAMI_high, toy_medium, toy_high	0.0181	14.270
CAMI_low, CAMI_high, toy_medium, toy_high	0.0318	16.050
CAMI_low, CAMI_medium, toy_medium, toy_high	0.0283	15.203
CAMI_low, CAMI_medium, CAMI_high, toy_high	0.0216	13.765
CAMI_low, CAMI_medium, CAMI_high, toy_medium	0.0209	14.078

Appendix D: Proof of upper bound on maximum chain length

We show that the maximum chain length within a cluster of size m is bounded above by $1 + \lfloor m/2 \rfloor$ using induction. First, note that this holds for $m = 1$: if a cluster contains one node, than that one node will directly point to the cluster ID and the chain has length 1. Now assume that the statement holds for clusters of size at most $m - 1$, that is, the longest chain in a cluster of size $m - 1$ is of length $1 + \lfloor (m-1)/2 \rfloor$. Consider a cluster of size m, which we denote as cluster A. This cluster was formed by merging two clusters, say clusters B and C with m_B and m_C nodes, $m_B \leq m/2$, $m_C \geq m/2$. Note that by assumption, since $m_C < m$, the length of the longest chain in cluster C is bounded by $1 + \lfloor m_C/2 \rfloor$. From these two clusters, we redirected the pointer of the head of the cluster with the shortest maximum chain, let's say that this chain has length l. The maximum chain in the new cluster has length $l + 1$ by construction. By assumption, the length of the maximum chain in cluster B does not exceed $1 + \lfloor m_B/2 \rfloor$, and thus $l \leq 1 + \lfloor m_B/2 \rfloor$. For $m = 2$ we have $m_B = m_C = 1$ and for $m = 3$ we have $m_B = 1$ and $m_C = 2$, which both gives $1 + \lfloor m_B/2 \rfloor = 1 = \lfloor m/2 \rfloor$. When $m \geq 4$ we can write:

$$1 + \lfloor m_B/2 \rfloor \leq 1 + \lfloor m/4 \rfloor \leq \lfloor m/2 \rfloor \, .$$

Hence for any $m > 1$ we have $l \leq \lfloor m/2 \rfloor$ and the maximum chain in the new cluster has a length of at most $1 + \lfloor m/2 \rfloor$.

Appendix E: Comparison of clustering methods on small datasets

Table S5. Clustering results for small read datasets obtained with MetaCluster 5.0, Abundancebin and OGRE. * Could not finish the clustering procedure within two months. MetaCluster 5.0 and Abundancebin allow the user to pre-specify the number of clusters. In the table, "known no. clusters" indicates the results obtained when providing the tool with the correct number of clusters, "unknown no. clusters" shows the results when the tool was not provided with a pre-specified number of clusters.

Dataset	Evaluation metric	MetaCluster 5.0 known no. clusters	MetaCluster 5.0 unknown no. clusters	Abundancebin known no. clusters	Abundancebin unknown no. clusters	OGRE
2species_a	% reads clustered for species ID 220	0	0	100	100	3.2
	% reads clustered for species ID 294	0.7	0.7	100	100	98.8
	No. clusters that contain species ID 220	0	0	2	1	56
	No. clusters that contain species ID 294	2	10	2	1	333
	Mean no. species per cluster [range]	1 [1,1]	1 [1,1]	2 [2,2]	2 [2,2]	1 [1,1]
	Total number of clusters	2	10	2	1	389
2species_b	% reads clustered for species ID 294	0.6	0.6	n.a.*	n.a.*	98.8
	% reads clustered for species ID 340	1.1	0.9	n.a.*	n.a.*	98.9
	No. clusters that contain species ID 294	1	4	n.a.*	n.a.*	332
	No. clusters that contain species ID 340	1	5	n.a.*	n.a.*	1
	Mean no. species per cluster [range]	1 [1,1]	1 [1,1]	n.a.*	n.a.*	1 [1,1]
	Total number of clusters	2	9	n.a.*	n.a.*	333
4species	% reads clustered for species ID 126	0.3	0.3	100	n.a.*	99.7
	% reads clustered for species ID 220	0	0	100	n.a.*	3.2
	% reads clustered for species ID 223	0	0	100	n.a.*	99.9
	% reads clustered for species ID 340	0.8	0.7	100	n.a.*	99.9
	No. clusters that contain species ID 126	1	1	4	n.a.*	25
	No. clusters that contain species ID 220	0	0	3	n.a.*	56
	No. clusters that contain species ID 223	0	0	4	n.a.*	2
	No. clusters that contain species ID 340	3	8	4	n.a.*	1
	Mean no. species per cluster [range]	1 [1,1]	1 [1,1]	3.75 [3,4]	n.a.*	1 [1,1]
	Total number of clusters	4	9	4	n.a.*	84

Appendix F: Computational performance

Table S6. Computational performance of OGRE.

Dataset	Runtime (CPU hours) Overlap graph construction	Clustering	Total	Size of the overlap graph Number of edges ($\times 10^9$)	Output file size (GB)
CAMI_mousegut	2358	243	2601	7.10	301
CAMI_low	2118	145	2263	8.10	310
CAMI_medium	1853	367	2220	7.04	360
CAMI_high	291	250	541	0.36	19
toy_medium	1762	291	2053	2.85	137
toy_high	648	24	672	2.33	108

Table S7. Performance of the logistic regression classifier. The training data for the dataset in column 1 is created by selecting 10,000 same species overlaps and 10,000 different species overlaps from each of the overlap graphs of the other four datasets. Test accuracy is obtained by applying the trained model to the overlap graph of the test dataset. The two right-most columns show the fraction of overlaps discarded by the logistic regression classifier.

Test dataset	Classification accuracies Train	Test	Fraction of overlaps discarded Same species overlaps	Different species overlaps
CAMI_low	0.715	0.801	0.199	0.935
CAMI_medium	0.775	0.923	0.077	0.446
CAMI_high	0.765	0.891	0.109	0.561
toy_medium	0.758	0.925	0.075	0.598
toy_high	0.772	0.934	0.066	0.483

picture(0,0)(-30,0)10 (-30,-5)(0,1)10 (-35,0)(1,0)30 (0,30)10 (-5,30)(1,0)10 (0,35)(0,-1)30 picturepicture(0,0)(30,0)10 (30,-5)(0,1)10 (35,0)(-1,0)30 (0,30)10 (-5,30)(1,0)10 (0,35)(0,-1)30 picture

Appendix G: Overlap length and Phred-based matching probability distributions

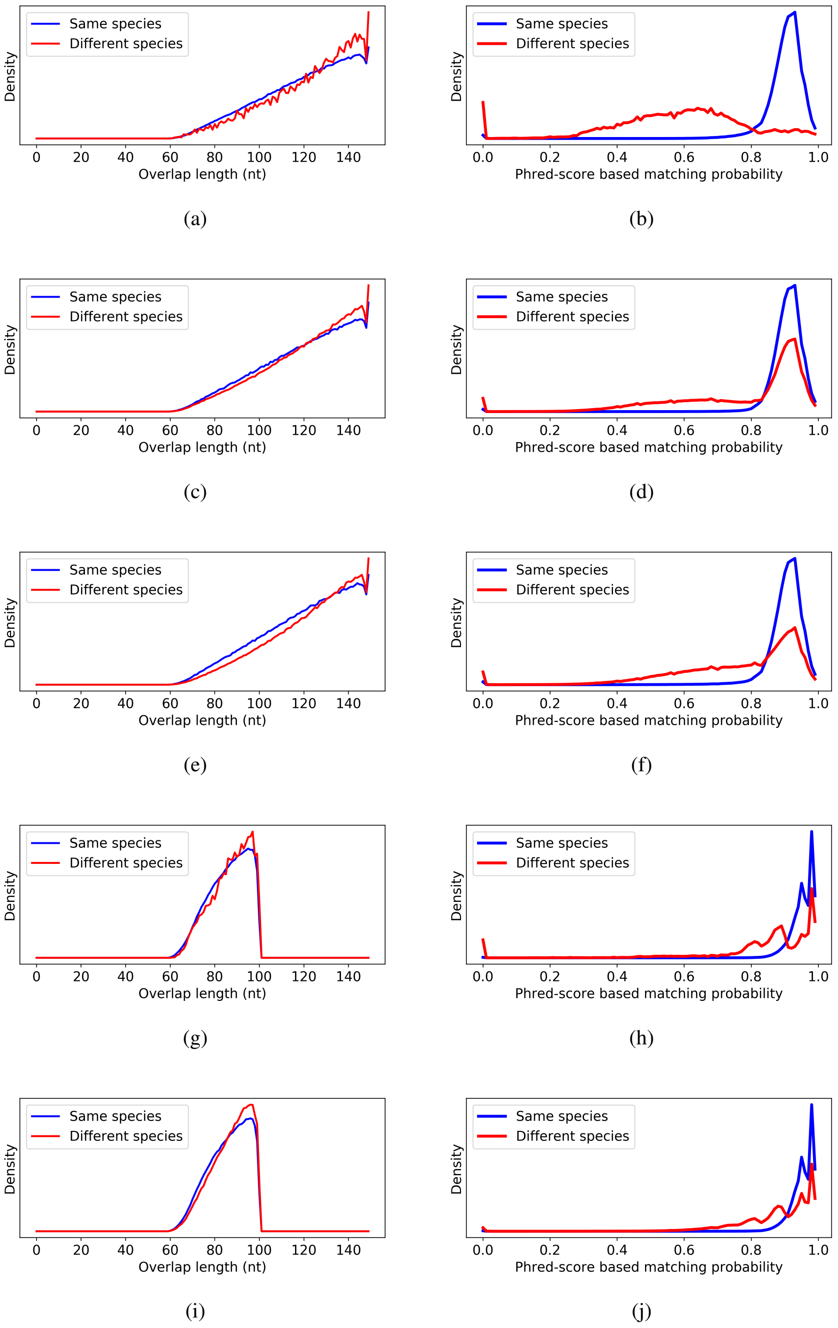

Fig. S1. Distribution of (a, c, e, g and i) the overlap length and (b, d, f, h and j) the Phred-based matching probability for 10,000 overlaps between reads from the same species and 10,000 overlaps between reads from different species selected from (a, b) CAMI_low, (c, d) CAMI_medium, (e, f) CAMI_high, (g, h) toy_medium and (i, j) toy_high. Note that two reads that are from the same species may originate from different strains.

picture(0,0)(-30,0)10 (-30,-5)(0,1)10 (-35,0)(1,0)30 (0,-30)10 (-5,-30)(1,0)10 (0,-35)(0,1)30 picturepicture(0,0)(30,0)10 (30,-5)(0,1)10 (35,0)(-1,0)30 (0,-30)10 (-5,-30)(1,0)10 (0,-35)(0,1)30 picture

Appendix H: Clustering results

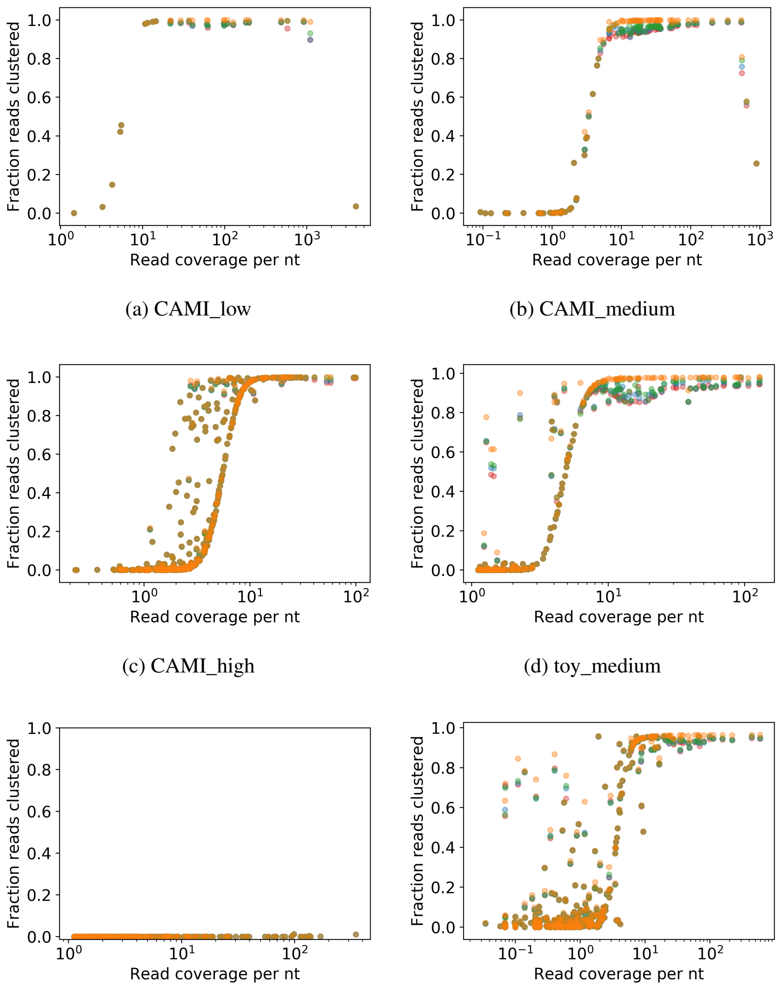

(a) CAMI_low

(b) CAMI_medium

(c) CAMI_high

(d) toy_medium

(e) toy_high

(f) mouse_gut

Fig. S2. Fraction of the reads that was clustered versus read coverage for (a) CAMI_low, (b) CAMI_medium, (c) CAMI_high, (d) toy_medium, (e) toy_high, and (f) mouse_gut. Each dot represents a species in the dataset. Results are presented for the four maximum allowed cluster sizes: 3,300 reads (red), 17,000 reads (blue), 33,000 reads (green) and no limit (orange). Note that for some species with extremely high coverage the number of clustered reads is low. These species are circular elements, which is something that Minimap2 has difficulties with.

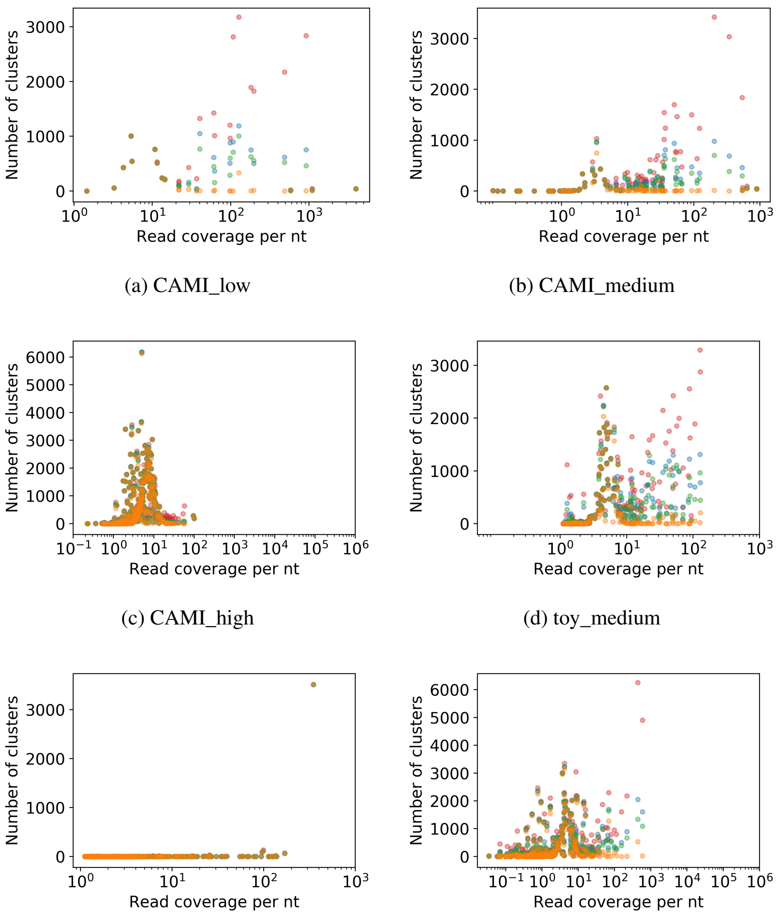

(a) CAMI_low (b) CAMI_medium

(c) CAMI_high (d) toy_medium

(e) toy_high (f) mouse_gut

Fig. S3. The number of clusters that contain at least one read from a species versus read coverage for (a) CAMI_low, (b) CAMI_medium, (c) CAMI_high, (d) toy_medium, (e) toy_high, and (f) mouse_gut. Each dot represents a species in the dataset. Results are presented for the four maximum allowed cluster sizes: 3,300 reads (red), 17,000 reads (blue), 33,000 reads (green) and no limit (orange).

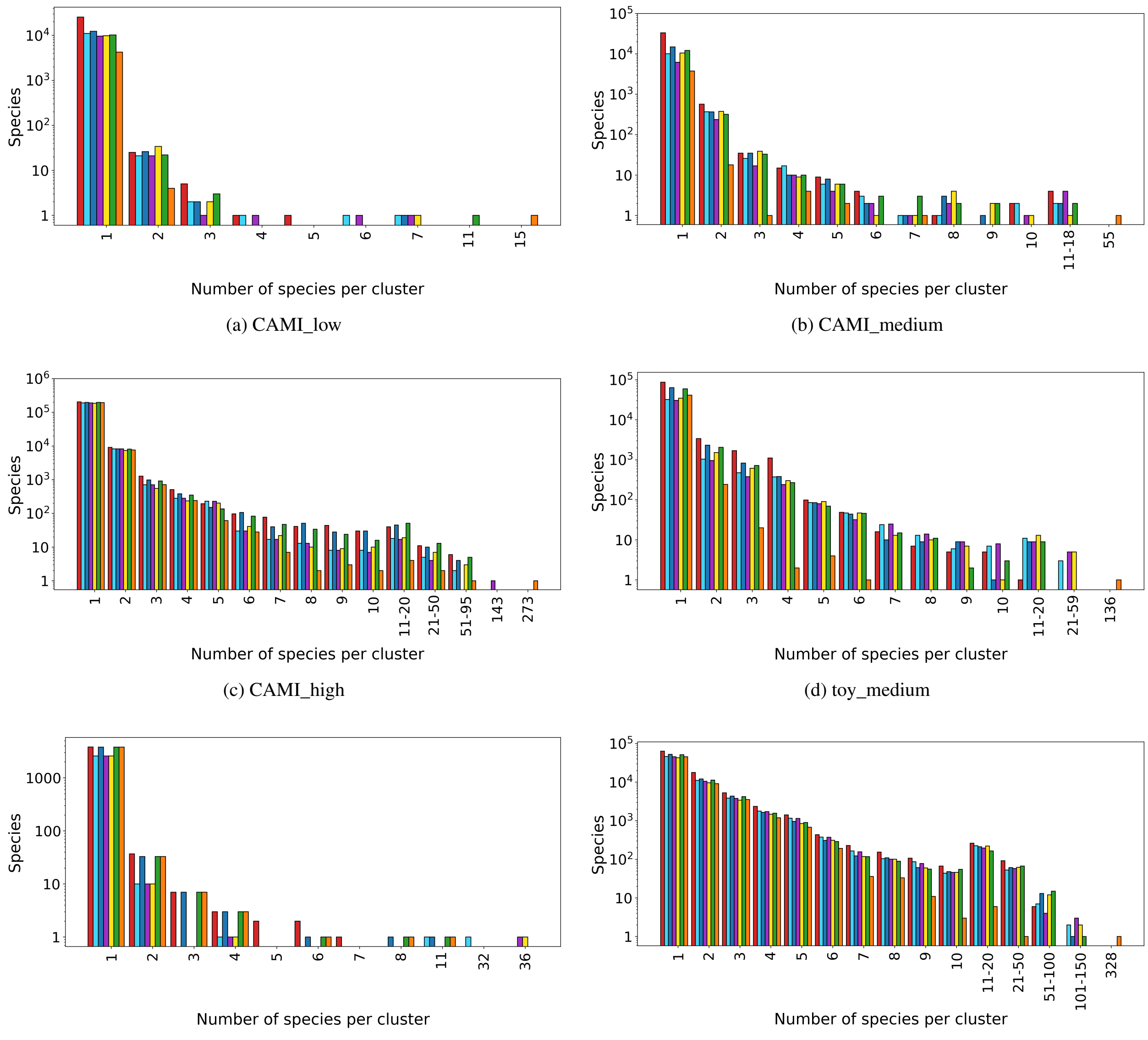

(a) CAMI_low

(b) CAMI_medium

(c) CAMI_high

(d) toy_medium

(e) toy_high

(f) mouse_gut

Fig. S4. Histograms of the number of species per cluster obtained with OGRE for a maximum cluster size of 3,300 reads (OGRE steps 1-3, red), 17,000 reads from 3,300 reads (OGRE steps 1-4, light blue), 17,000 reads (OGRE steps 1-3, dark blue), 33,000 reads from 3,300 reads (OGRE steps 1-4, purple), 33,000 reads from 17,000 reads (OGRE steps 1-4, yellow), 33,000 reads (OGRE steps 1-3, green) and unlimited (OGRE steps 1-3, orange). Results are shown for (a) CAMI_low, (b) CAMI_medium, (c) CAMI_high, (d) toy_medium, (e) toy_high, and (f) mouse_gut.

Appendix I: Genes that reads from multi-species clusters map to

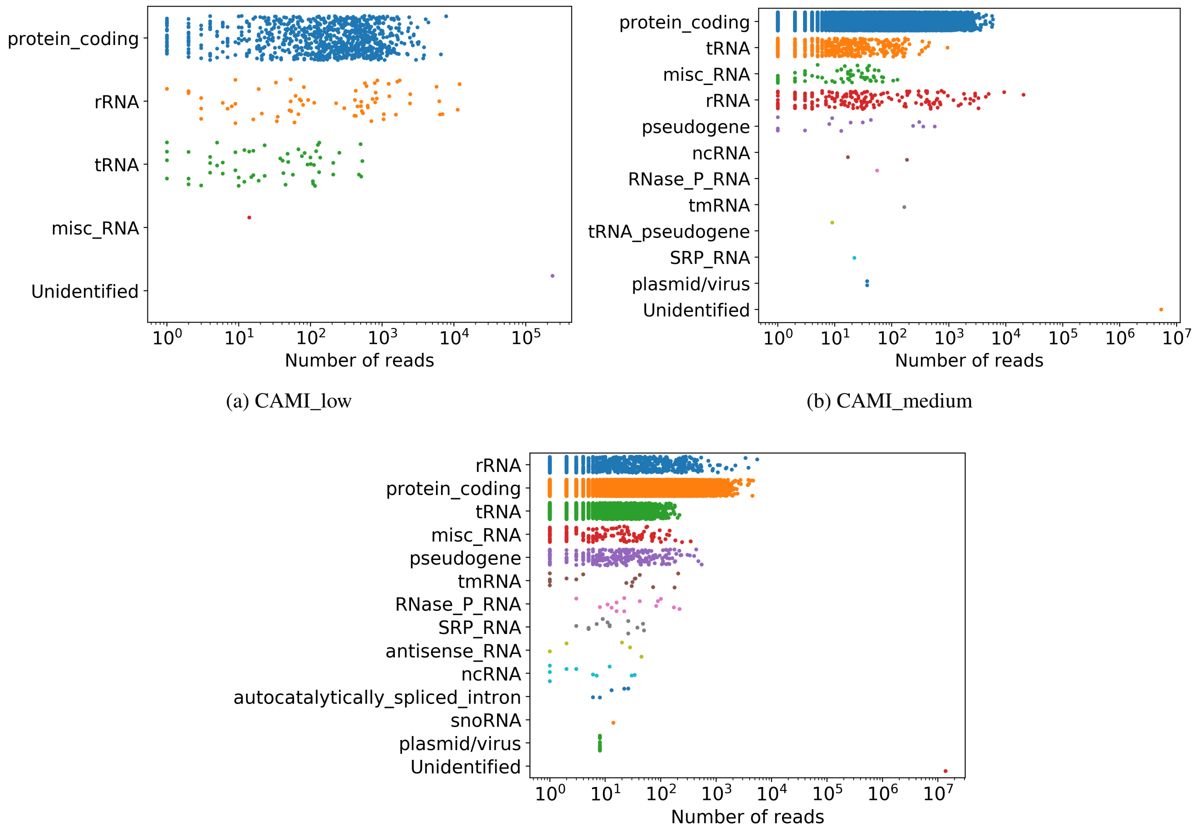

(a) CAMI_low

(b) CAMI_medium

(c) CAMI_high

Fig. S5. Reads from all clusters that contain at least two species were mapped to the source genome. This figure shows the type of genes that these reads map to.

Appendix J: Assembly results

Table S8. Assembly results for SPAdes (**?**) applied to the original data without clustering (“None”) and applied to clustered data. Two clustering approaches were compared: a random clustering with the same number of clusters as OGRE came up with, and a clustering obtained with OGRE. SPAdes was applied to clustered data using a three-step approach: (1) cluster reads, (2) assemble the reads for each cluster separately, and (3) assemble the reads using SPAdes where the contigs obtained in step (2) are used as a guide. Partially unaligned length is the number of unaligned bases in those contigs that were only partially aligned with the reference genome.

	CAMI_low			CAMI_medium			CAMI_high			CAMI_mousegut		
Clustering method	None	Random	OGRE	None	Random	OGRE	None	Random	OGRE	None	Random	OGRE
Genome fraction (%)	78.5	82.0	79.6	58.8	59.6	60.4	54.9	56.2	55.2	38.4	38.7	38.7
N50	50,399	51,155	69,015	32,857	30,625	72,244	2,549	2,572	3,508	6,719	6,528	9,908
NA50	46,136	46,974	60,749	30,483	28,508	63,764	2,355	2,399	3,294	4,367	4,303	5,778
# Misassemblies	874	970	854	3,993	4,240	3,138	85,113	77,223	72,123	36,700	35,912	34,589
# Mismatches per 100 kbp	327.0	449.8	355.1	386.5	415.3	375.2	922.1	989.2	960.3	759.1	779.0	780.2
Partially unaligned length	4,574	4,106	698	13,486	14,454	18,149	797,245	783,572	630,866	16,752,856	16,378,152	19,090,321
